# Endovascular Management of Right Subclavian Artery Pseudoaneurysm due to War Injury in Adolescent Patient

**DOI:** 10.1155/2017/9030457

**Published:** 2017-09-11

**Authors:** Onur Saydam, Deniz Şerefli, Mehmet Atay, Cengiz Sert

**Affiliations:** ^1^Tepecik Training and Research Hospital, Department of Cardiovascular Surgery, 35170 İzmir, Turkey; ^2^Bakirkoy Dr. Sadi Konuk Training and Research Hospital, Department of Cardiovascular Surgery, 34147 İstanbul, Turkey

## Abstract

Today there is a widespread use of endovascular treatment (EVT) for traumatic vascular injuries in adults, but there is lack of evidence of its use in adolescent patients with vascular injuries. With this case, we present successful EVT of 14-year-old adolescent with a right subclavian artery pseudoaneurysm (SAP) due to war injury. SAP was successfully excluded with deployment of 6 × 50 mm flexible, self-expanding covered nitinol stent graft (The GORE® VIABAHN® Endoprosthesis (W.L. Gore & Associates, Flagstaff, AZ)). Patient was discharged from hospital 2 days after the procedure with dual antiplatelet therapy (clopidogrel and aspirin). 3 months after discharge control DUS showed patent stent graft without any residual lesions. As a result, EVT is an alternative approach to treatment of SAP. It is safe, effective, and less invasive therapy for SAP in adults as well as in adolescents. We aim to contribute to the literature with this first case report.

## 1. Introduction

Nowadays war related injuries have begun to appear with increase in frequency in Turkey due to state of war in neighboring countries. The usage of firearms and explosive weapons in these situations can cause vascular injuries [1]. Subclavian artery pseudoaneurysm (SAP) or rupture is not common but can occur due to clavicle fracture, firearms, and other trauma [2]. Because of close relation to vital structure and noncompressibility, SAP remains a challenging problem for surgeons. Currently less invasive techniques rather than open surgery (OS) are being discussed for SAP in adult patients [3, 4]. However, there is lack of evidence of usage of endovascular therapy (EVT) in adolescents suffering SAP. We report a case of an adolescent with traumatic SAP, which was formed secondary to war injury and was successfully treated by EVT.

## 2. Case Report

**Figure 1:**
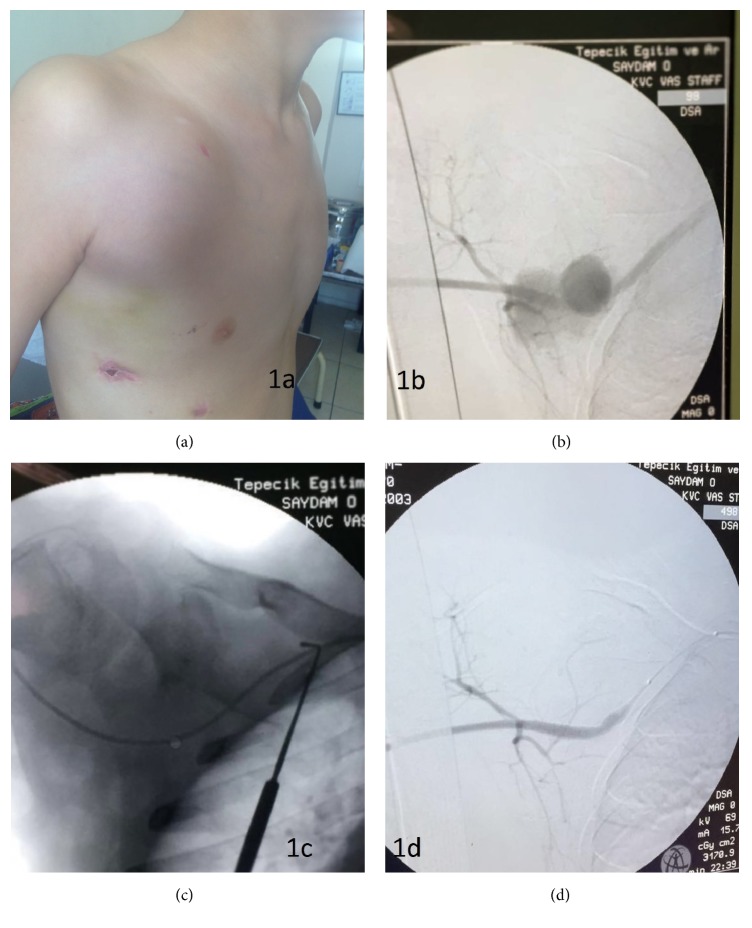
(a) Large subclavian pseudoaneurysm pulsatile sac with palpable trill. (b) Angiogram of pseudoaneurysm sac originated from the right subclavian artery. (c) Passing the lesion with 0.035 hydrophilic guide wire with the help of 5F straight selective multipurpose diagnostic catheter. (d) Final selective angiogram of the right subclavian artery with a complete hemostasis and preserved subclavian artery branches.

A 14-year-old boy was admitted to emergency department with complaint of pain in right upper pectoral area. Physical examination revealed a wound scar with a diameter of 0.5 × 0.2 mm and a pulsatile mass with a diameter of 6 × 6 cm in the right upper pectoral area. There was also a scar related to thorax drainage tube in the mid-clavicular line, which was placed 2 months before in a hospital right after the explosion in Syria (Figure 1(a)). The reason of placement of drainage tube remained unknown due to lack of medical records. Anamnesis revealed that the pulsatile mass was growing with each passing day. He was hemodynamically stable. Right upper extremity pulses were palpable and no signs were observed pointing to circulatory disorder. Color Doppler Ultrasonography (DUS) showed a large SAP (5 × 5 cm) with a yin yang sign. SAP was originating from right subclavian artery (RSA) and it was approximately 2.5 mm wide. Contrast enhanced computed tomography angiography was performed to evaluate the vascular dimensions. Proximal RSA diameter was 5.3 mm and distal RSA was 5.2. We preferred EVT and performed the procedure under general anesthesia. Heparin was administered to maintain activated coagulation time above 250 seconds. Retrograde percutaneous approach from the right femoral artery to RSA with insertion of 5F vascular sheath into common femoral artery was performed. Selective angiography of the RSA with 5F pigtail catheter via left anterior oblique view with an angle 25° showed an SAP (Figure 1(b)). We passed the lesion with 0.035 hydrophilic guide wire with the help of 5F straight selective multipurpose diagnostic catheter (Figure 1(c)). We removed 5F vascular sheath and exchanged with 7F vascular sheath. SAP was excluded with deployment of 6 × 50 mm flexible, self-expanding covered nitinol stent graft (GORE VIABAHN Endoprosthesis (W.L. Gore & Associates, Flagstaff, AZ)). Postdilation inside of the stent graft with balloon angioplasty was performed. We preserved the RSA branches (Figure 1(d)). Control angiography showed SAP was completely excluded and there was no extravasation. Blood flow of the upper extremity was normal. 7F vascular sheath was removed and manual compression was applied to vascular access site. He was extubated in the operation room. The total duration of the operation was 20 minutes. No loading dose was given for clopidogrel and aspirin. The treatment was started right after the procedure with 1 mg/kg clopidogrel and 2 mg/kg of aspirin. 0.01 cc/kg enoxaparin was administered 2 hours after the operation. The patient discharged from hospital on postoperative day 2 without any vascular access site related complication. 3 months after discharge control DUS showed patent stent graft without any residual lesions.

## 3. Discussion

Today there is a widespread use of endovascular treatment for traumatic vascular injuries in adults, but there is lack of evidence of its use in adolescent patients with vascular injuries. With this case report, we aim to contribute to the literature with this first case report, to the best of our knowledge in adolescent patients with penetrating SAP caused by war injury and treated with EVT. Although there is a case report about EVT of SAP in adolescent patient with Ehlers-Danlos syndrome (EDS), before the trauma our patient was totally healthy [5]. EDS is a dominantly inherited connective tissue disorder and EDS type 4 (the vascular type) is mainly caused by a deficit of type 3 fibrillar collagen which is constituent of arterial wall [6]. Differently from EDS patients we accept that our patient has healthy arterial wall and this may lead to difference in results. RSA arises from brachiocephalic artery and extends to the lateral border of the first rib. RSA injuries can be seen 5–10% percent in the population and most of these injuries are caused by penetrating traumas [7]. OS or EVT can be the treatment strategies for SAP. OS is usually safe but because of the injury risk of adjacent structures during surgical exploration complication rates are reported to be up to 24% in OS [8]. According to studies, depending on hemodynamic stability at presentation, mortality rates could range from 5% to 30% [9, 10]. Because of the significant morbidity and mortality rates, we preferred EVT rather than OS. Also, in some cases, thoracotomy may be required to obtain proximal control of the artery and in our case possible adhesions due to tube replacement might further complicate the procedure. There are some handicaps for EVT in adolescent population. Vascular access is one of these problems. The brachial artery is mostly used in adult patients, as it provides a direct, shorter, and less tortuous approach [11]. In our patient after DUS evaluation we have chosen femoral retrograde approach rather than RSA because of the need for the use of 7F vascular sheath in order to introduce stent graft. The other problem for usage of EVT in adolescents is the diameter of the target vessel because of continuing vessel growth. In our case, the diameter of RSA was 5.2 mm and because RSA is mobile and can be exposed to rotational forces during abduction and anteflexion of the arm we preferred to use 6 mm × 50 mm GORE VIABAHN Endoprosthesis (W.L. Gore & Associates, Flagstaff, AZ) rather than bare-metal stent. It is a nitinol supported flexible, self-expanding stent graft made of an expanded polytetrafluoroethylene. Unlike the bare-metal self-expanding stents we did not oversize VIABAHN stent. Because according to studies oversizing the VIABAHN stents by more than 20% of the vessel diameter will end up with significantly lower patency rates [12]. A study revealed that stent sizes of 6 mm and above for RSA would keep the patient asymptomatic in adult population [13]. Patency is an important issue for stent grafts. There are studies on this stent graft which show good patency rates [14, 15]. Postprocedural management is also having positive impact on patency of the graft. The optimal duration of dual antiplatelet therapy with aspirin and clopidogrel after endovascular treatment remains controversial [16, 17]. Dual antiplatelet therapy with aspirin and clopidogrel with loading dose is mostly recommended for 6 months in adults. There are also studies suggesting more aggressive and longer antithrombotic regimens after VIABAHN stent graft placement [18]. Because of lack of evidence for loading dose of clopidogrel in adolescent we did not use loading dose and because of long life expectancy we planned to give clopidogrel for 6 months and 100 mg aspirin therapy for life long. Although all these were taken into consideration there are some studies that show 16.7%–34% thrombosis rates [19, 20]. Being different than SAP, these studies are mostly made on occlusive disease which is progressing disease and may not have adequate inflow and outflow and this situation can affect the patency rates of the graft. Nevertheless, stent thrombosis does not preclude future revascularization, which, if necessary, can be done under less emergent circumstances after the acute injury has resolved [21].

## 4. Conclusion

As a result, EVT is an alternative approach to treatment of SAP. It is safe and effective as well as less invasive therapy for SAP. It can also be performed safely in adolescent patients. Periodic patient follow-ups with DUS and postprocedural therapy strategies may have a positive impact on patency. But there is still lack of knowledge for long-term effectiveness and patency.
